# X-Linked Genes and Risk of Orofacial Clefts: Evidence from Two Population-Based Studies in Scandinavia

**DOI:** 10.1371/journal.pone.0039240

**Published:** 2012-06-19

**Authors:** Astanand Jugessur, Øivind Skare, Rolv T. Lie, Allen J. Wilcox, Kaare Christensen, Lene Christiansen, Truc Trung Nguyen, Jeffrey C. Murray, Håkon K. Gjessing

**Affiliations:** 1 Division of Epidemiology, Norwegian Institute of Public Health, Oslo, Norway; 2 Craniofacial Research, Murdoch Childrens Research Institute, Royal Children’s Hospital, Parkville, Australia; 3 Department of Public Health and Primary Health Care, University of Bergen, Bergen, Norway; 4 Medical Birth Registry of Norway, Norwegian Institute of Public Health, Bergen, Norway; 5 Epidemiology Branch, National Institute of Environmental Health Sciences (National Institute of Health, Durham, North Carolina, United States of America; 6 Department of Epidemiology, University of Southern Denmark, Odense, Denmark; 7 Department of Clinical Biochemistry and Pharmacology, Odense University Hospital, Odense, Denmark; 8 Department of Clinical Genetics, Odense University Hospital, Odense, Denmark; 9 Departments of Pediatrics, Epidemiology and Biological Sciences, University of Iowa, Iowa City, Iowa, United States of America; University of Montreal, Canada

## Abstract

**Background:**

Orofacial clefts are common birth defects of complex etiology, with an excess of males among babies with cleft lip and palate, and an excess of females among those with cleft palate only. Although genes on the X chromosome have been implicated in clefting, there has been no association analysis of X-linked markers.

**Methodology/Principal Findings:**

We added new functionalities in the HAPLIN statistical software to enable association analysis of X-linked markers and an exploration of various causal scenarios relevant to orofacial clefts. Genotypes for 48 SNPs in 18 candidate genes on the X chromosome were analyzed in two population-based samples from Scandinavia (562 Norwegian and 235 Danish case-parent triads). For haplotype analysis, we used a sliding-window approach and assessed isolated cleft lip with or without cleft palate (iCL/P) separately from isolated cleft palate only (iCPO). We tested three statistical models in HAPLIN, allowing for: i) the same relative risk in males and females, ii) sex-specific relative risks, and iii) X-inactivation in females. We found weak but consistent associations with the oral-facial-digital syndrome 1 (*OFD1*) gene (formerly known as *CXORF5*) in the Danish iCL/P samples across all models, but not in the Norwegian iCL/P samples. In sex-specific analyses, the association with *OFD1* was in male cases only. No analyses showed associations with iCPO in either the Norwegian or the Danish sample.

**Conclusions:**

The association of *OFD1* with iCL/P is plausible given the biological relevance of this gene. However, the lack of replication in the Norwegian samples highlights the need to verify these preliminary findings in other large datasets. More generally, the novel analytic methods presented here are widely applicable to investigations of the role of X-linked genes in complex traits.

## Introduction

Orofacial clefts are relatively common craniofacial birth defects, with a birth prevalence of about 1–2/1000. They require extensive surgical, nutritional, dental, speech, behavioral and medical interventions, and thus impose a substantial economic and personal health burden [1], [2]. As with other complex traits, multiple genetic and environmental risk factors are thought to underlie these birth defects [3].

**Table 1:** Review of family-based methods for association analysis of X-chromosome markers.

Reference	Method	Extended name	Attributes
Ho and Bailey-Wilson[23]	X-TDT	X-linkage transmission/disequilibrium test (TDT)	This is a TDT for linkage on the X chromosome in the presence of linkage disequilibrium (LD). Under H_o_ of no linkage between disease and marker, the number of transmissions of the variant allele in *n* pairs of heterozygous mothers and their affected children has a binomial distribution with mean *n*/2 and variance *n*/4. The test statistic is a Z-score with a continuity correction, and H_o_ is rejected if Z departs significantly from 0. As with TDT, X-TDT is readily extended to allow the analysis of phenotypically discordant sib pairs if parental genotypes are unavailable (suitable for late-onset diseases). It can also combine sib-pair and case-parent triad analysis to enhance statistical power.
Horvath et al. [21];Knapp [22]	XS-TDT;XRC-TDT	X-linked sibling TDT; Reconstruction-combinedTDT for X-chromosomemarkers	As X-TDT above, these are tests for linkage between an X-chromosomal marker and a disease in the presence of LD. XS-TDT uses the genotypes of discordant sibships if genotypes are not available from the parents. It divides the siblings into same-sex groups to account for a possible male/female difference in disease prevalence. XRC-TDT reconstructs parental genotypes from the genotypes of their offspring and corrects for bias that arise from the reconstruction. Data from families in which parental genotypes are available are combined with families in which genotypes of unaffected sib pairs are available.
Ding et al. [24]	XPDT;XMCPDT	X-chromosomal pedigree disequilibrium test;Monte Carlo pedigreedisequilibrium test for X-linkedmarkers	XPDT tests for LD in the presence of linkage. It can be applied to any pedigree structure. XMCPDT is an extension of XPDT and infers missing parental genotypes using a Monte Carlo sampling approach. XPDT is limited to same-sex discordant sib pairs when parental data are missing, resulting in lower statistical power. XMCPDT on the other hand requires allele frequency estimates to compensate for missing parental genotypes. XMCPDT appears to have superior power than XSTDT, XRCTDT or XPDT when there are missing data, but Type 1 errors can be inflated when a large proportion of parental genotypes are missing.
Chung et al. [25]	X-APL	A modification of the“association in thepresence of linkage test(APL)=" that accommodatesX-chromosomemarkers	Like XPDT, X-APL can use singleton or multiplex families. The APL statistic is based on difference between the observed versus the expected number of a specific allele in affected siblings conditional on the parents’ genotypes. X-APL infers missing parental genotypes in linkage regions by using identity-by-descent (IBD) parameters for affected siblings. X-APL can test individual markers or haplotypes. For haplotype tests, X-APL assumes no recombination between the markers within the families in the sample, and the EM algorithm is used for haplotype phase estimation. X-APL can also perform sex-stratified analyses to account for different penetrance of disease in males versus females.
Zhang et al. [27]	X-LRT	A likelihood ratio test ofassociation for X-linkedmarkers.	X-LRT is a likelihood-based method and enables estimation of genetic risks. The method is designed for singleton families but can also allow additional siblings. Missing parental genotypes can be accounted for using the EM algorithm, and even more efficiently using sibling genotype information when available. For haplotype relative risk estimation, X-LRT assumes no recombination between markers, parental mating to be random, and haplotype penetrance to be multiplicative for females. For sex-specific analysis, separate risk parameters are introduced for males and females in single-marker analyses, but in haplotype analyses the data are divided into two sets, one containing only male cases and the other only female cases.
This paper	HAPLIN	A full likelihood model forhaplotype associationsat autosomal and X-linkedmarkers.	HAPLIN is a likelihood-based method and enables estimation of genetic risk associated with marker haplotypes both for autosomal and X-linked markers. It applies to case-parent triad data, possibly combined with independent controls and/or complete control-parent triads. Missing data are imputed using the EM algorithm. On the X chromosome, HAPLIN provides a range of model options depending on haplotype effects in females versus males. A complete sex stratification implies different haplotype frequencies, different baseline risks and different relative risks between males and females. Alternatively, haplotype frequencies can be assumed equal, as can haplotype relative risks. The risk response pattern may depend on the number of risk haplotypes, and X-inactivation in females can be incorporated.

**Table 2:** Sample distribution according to cleft type, sex, and population.

	Norway		Denmark	
Cleft type	Males	Females	Males	Females
iCL/P	202	109	114	52
iCPO	54	60	33	36

Clefts are characterized by a particularly strong genetic component, as evidenced by studies of familial recurrence risk and heritability [4]. First-degree relatives of an affected individual have a 30–40 fold higher recurrence risk compared with the background population [5], [6], and heritability estimates were recently reported to be as high as 91% for CL/P and 90% for CP in a large Danish twin study [7].

The strong genetic component to clefting has spurred decade-long efforts to identify the genes underpinning these complex birth defects. Increased collaborative efforts coupled with major advances in high-density SNP genotyping arrays have heralded a new era of gene discovery for complex traits. For clefts, the first genome-wide association study (GWAS) identified a strong signal on chromosome 8q24 in individuals of central European ancestry. This association was subsequently replicated in three independent GWAS [8]–[10]. In addition to the 8q24 locus, these studies also identified significant associations with several genes, including v-maf musculoaponeurotic fibrosarcoma oncogene homolog B (*MAFB*), ATP-binding cassette sub-family A member 4 (*ABCA4*), ventral anterior homeobox 1 (*VAX1*), paired box 7 (*PAX7*) and interferon regulatory factor 6 (*IRF6*) [4]. *IRF6* is particularly noteworthy, being the only gene on this list to be confirmed as a major player for clefts through approaches other than GWAS [11]–[13].

The above studies and virtually all association studies of clefts (as well as other complex traits) have targeted primarily autosomal markers, without attention to potential contributions of X-linked common gene variants. This is partly because most of the statistical methods originally designed for association analysis were only targeted towards the analysis of autosomal markers. The finding that X-linked gene variants may be implicated in a number of complex traits [14]–[19] has encouraged the development of statistical methods for analyzing X-linked markers. The majority of these methods are extensions of the transmission/disequilibrium test (TDT) [20], for example, the X-linked sibling TDT (XS-TDT) [21], the reconstruction-combined TDT for X-chromosome markers (XRC-TDT) [22], the X-linkage TDT (X-TDT) [23], and the X-chromosome pedigree disequilibrium test (XPDT) [24]. Two additional tests compare observed versus expected distributions of a specific allele or haplotype in affected siblings, conditional on the parental genotypes. These are the 1) association in the presence of linkage (APL) test that accommodates X-chromosome markers (X-APL) [25], and 2) X-linked quantitative trait loci linkage mapping (X-QTL) [26]. Despite several attractive attributes of these methods (summarized in **Table 1**), an important limitation is that they can only provide a p-value for association, but not estimates of genetic risk. The exception is the likelihood ratio test (LRT) developed by Zhang and co-workers (X-LRT) [27].

An exploration of X-linked variants is particularly relevant when a complex trait is more common in one sex – as is seen for the two main types of orofacial clefts. For this study, we implemented new functionalities in the HAPLIN software [28] to enable X-chromosome marker analysis and an estimation of relative risks associated with either a single or double dose of an allele or haplotype. We considered various model parameterizations that address a range of causal scenarios relevant to an X-linked disease locus. This included allowing for different baseline risks for males and females (to reflect the higher prevalence of CL/P in males), and accounting for X-inactivation in females (where one of the two copies of the X chromosome is inactivated in each cell to ensure similar gene dosage across the two sexes [29]).

**Table 3:** Assorted parameterization models for analysis of X-linked gene variants using the HAPLIN software.

Model	Male case		Female case		
	X_1_	X_2_	X_1_X_1_	X_1_X_2_	X_2_X_2_
Model 1	B	B*RR	B	B*RR	B*RR^2^
Model 2	B_M_	B_M_*RR	B_F_	B_F_*RR	B_F_*RR^2^
Model 3	B_M_	B_M_*RR_M_	B_F_	B_F_*RR_F_	B_F_*RR_F_^2^
Model 4	B_M_	B_M_*RR	B_F_	1/2*B_F_*(1+RR)	B_F_*RR
Model 5	B_M_	B_M_*RR_M_	B_F_	B_F_*RR_F1_	B_F_*RR_F2_

X_1_ denotes the common allele and X_2_ the variant or target allele for a given SNP; ‘*’ denotes the product term; B represents the shared baseline risk for males and females; B_M_ is the baseline risk for males only; B_F_ is the baseline risk for females only; RR is the shared relative risk for males and females; RR_M_ is the relative risk for males only; and RR_F_ is the relative risk for females only. In Model 4, the risk for an X_1_X_2_ female is the average of the two homozygotes; i.e. (B_F_+B_F_*RR)/2 = B_F_(1+RR)/2. As this is not a log-linear model, HAPLIN replaces the heterozygous risk with B_F_√RR, i.e. the geometric mean of the two homozygous risks. Models 3 and 5 can be estimated assuming equal or unequal haplotype frequencies between males and females.

We applied this new method to a collection of 48 SNPs in 18 cleft candidate genes on the X chromosome and used data from two national cleft studies in Scandinavia – one of the largest collections of orofacial cleft triads available. To our knowledge, no previous study has explored the role of X-linked genes in the etiology of orofacial clefts using association.

## Materials and Methods

### Study Populations, Candidate Genes and SNPs

From a population-based case-control study of orofacial clefts in Norway (1996–2001), 311 iCL/P and 114 iCPO case-parent triads were available for the current analysis. As a replication sample, we had a further 166 iCL/P and 69 iCPO case-parent triads from a population-based study of orofacial clefts in Denmark (1991–2001). The sample distribution according to cleft type, sex, and population is provided in **Table 2**. Details regarding study design and participants have been provided elsewhere [30], [31]. The 18 X-linked genes and 48 SNPs for the current analysis derive from a larger candidate-gene based study in which we examined 357 candidate genes in the same study populations [32]. A detailed description of these 18 X-linked genes and 48 SNPs is provided in the online **Table S1**.

**Figure 1:**
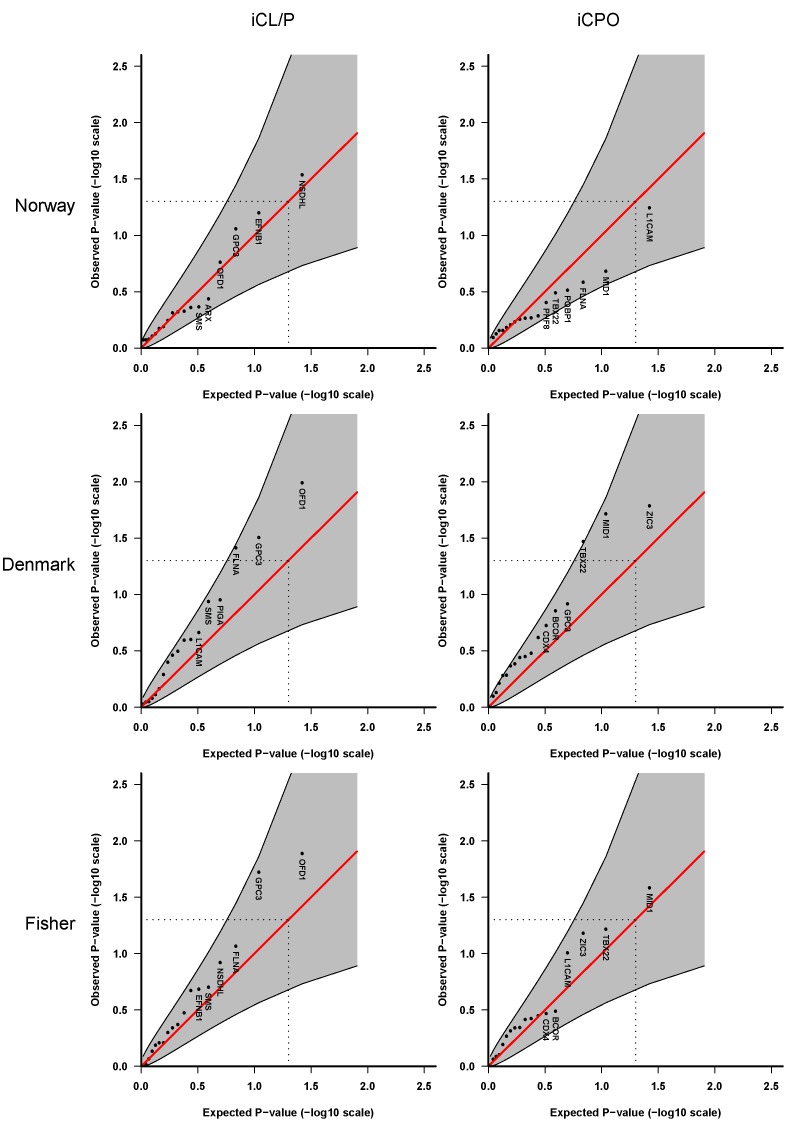
Single-marker analyses of 48 SNPs in 18 X-linked cleft candidate genes. These analyses are based on **Model 2** in which we assume different baseline risks for males and females, a shared relative risk for males and females, and no X-inactivation. Quantile-quantile (QQ) plots of p-values for iCL/P (left-hand side) and iCPO (right-hand side). Top panels: Norwegian and Danish samples, respectively. Bottommost panels: Fisher combined p-values. Shaded areas represent 95% confidence interval bands and dotted lines indicate the expected ranked p-value of 0.05. Note that the oral-facial-digital syndrome 1 gene (*OFD1*) was formerly known as *CXORF5*.

**Figure 2:**
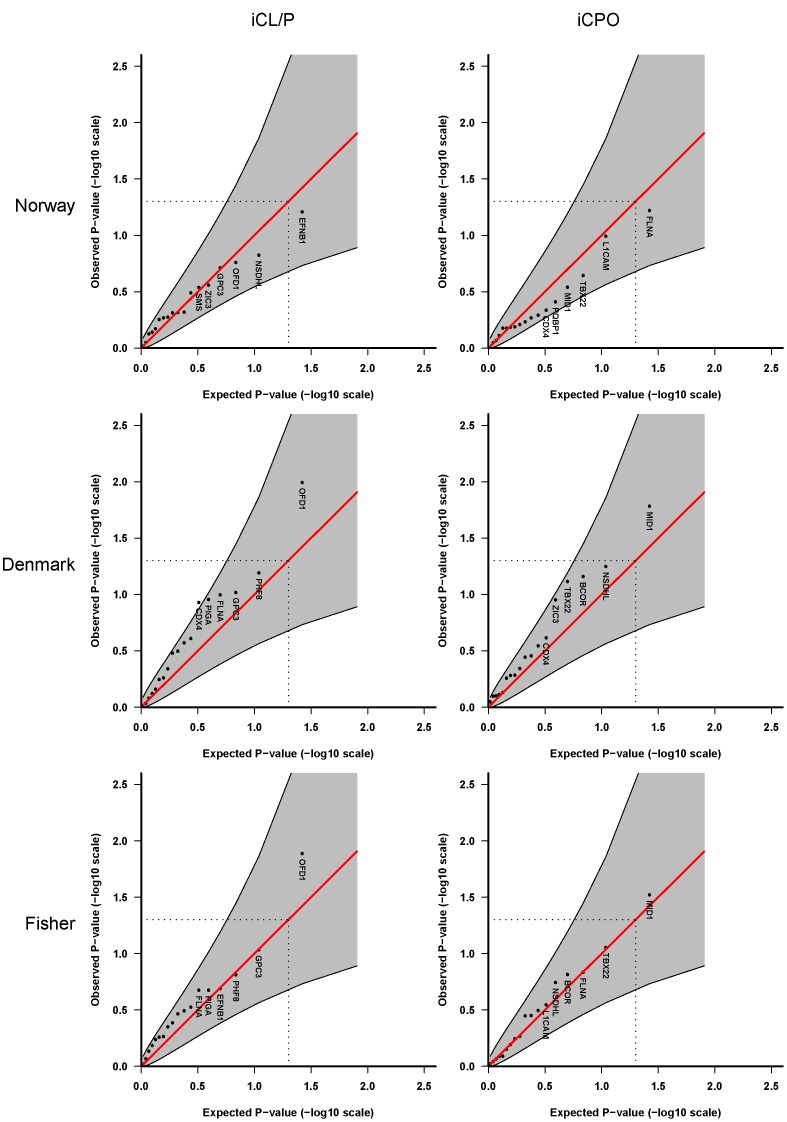
Haplotype analyses using up to 4 SNPs per sliding-window, Model 2.

### Statistical Analysis

#### The HAPLIN software

The statistical software HAPLIN [28] was specifically designed to analyze genetic and environmental risk factors in offspring-parent triads and case-control collections. It is based on log-linear modeling as originally described [33]–[41] and implements a full maximum-likelihood model for estimation. HAPLIN computes explicit estimates of relative risks with asymptotic standard errors and confidence intervals. It uses the expectation-maximization (EM) algorithm to impute genotypes that are missing at “random=" (e.g. due to failed genotyping) and those missing by “design=" (e.g. if DNA from a family member was not available for genotyping). The EM algorithm can also reconstruct unknown haplotype phase for haplotype analysis, but on the X chromosome this is not needed since phase can be deduced directly when data are non-missing.

Central to HAPLIN is a generalized linear model (glm) being estimated from the observed genotype frequencies–the M-step of the EM algorithm. The E-step consists of all three imputations described above, performed in a single step. The algorithm then alternates between the M-step and E-step until convergence is achieved. The results obtained from the EM algorithm correspond to the maximum-likelihood estimates of the model, which include gene frequencies and all relative risk parameters. However, to obtain the correct standard errors, confidence intervals and likelihood ratio test (LRT) for the models, HAPLIN corrects for the fact that imputation has taken place. If the imputed data were used uncorrected, they would seem to contain more information than what is actually available in the raw data.

#### X-linked haplotype analysis using HAPLIN

HAPLIN allows a range of X-chromosome models to be estimated, depending on assumptions made about allele effects in males versus females. The following models, summarized in **Table 3**, are examples of risk parameterizations provided by HAPLIN:

- **Model 1**: A shared baseline risk for males and females; a shared relative risk for males and females; no X-inactivation; a multiplicative dose-response relationship in females (1 risk parameter to be estimated).
- **Model 2**: Different baseline risks for males and females; a shared relative risk for males and females; no X-inactivation; a multiplicative dose-response relationship in females (2 risk parameters to be estimated)
- **Model 3**: Different baseline risks for males and females; different relative risks for males and females; no X-inactivation; a multiplicative dose-response relationship in females (3 risk parameters to be estimated)
- **Model 4**: Different baseline risks for males and females; a shared relative risk for males and females; X-inactivation; a multiplicative dose-response relationship in females (2 risk parameters to be estimated)
- **Model 5**: Different baseline risks for males and females; different relative risks for males and females; no assumption of multiplicative risks. We refer to this as the “free model=", with the highest number of parameters to be estimated (4 risk parameters).

With fewer parameters, more assumptions are needed but power is improved (provided the model is correct). The log-linear model implemented in HAPLIN extends the X-LRT approach described by Zhang and colleagues [27], which essentially estimates **Model 5** for single SNP markers, although with a different parameterization. Even though stratifying the case-parent triads by sex may reduce the statistical power to detect an association, we performed sex-specific analyses to verify whether there is a stronger gene effect in one sex versus the other. **Model 1**, with the assumption of equal baselines, is less relevant to our data. **Models 3** and **5** can be estimated assuming equal allele frequencies between males and females, or by completely stratifying on sex. All models have natural extensions from single SNP markers to testing multiple haplotypes.

#### Sliding windows and multiple testing

Our study comprised one of the largest collections of case-parent triads for clefts from two population-based sources. Even so, it is unlikely that gene-effects are large enough that a single gene would remain significant after a full Bonferroni correction, even when the two sexes are analyzed together. The stringent requirement of ensuring an overall Type 1 error rate of ≤5% will be overly conservative, especially in a study such as this one where the candidate genes had been selected *a priori* for their potential roles in clefting.

To adequately deal with the multiple-testing issue, we followed a two-part approach. First, all p-values computed from single SNPs or haplotypes within a gene were summarized into a single p-value for that gene, corrected for within-gene multiple testing. Second, the single gene p-values were plotted together in a quantile-quantile (QQ) plot, which would reveal p-values more significant than what would be expected by chance given the number of genes being tested.

For the first part, HAPLIN includes the function haplinSlide (for details, see http://www.uib.no/smis/gjessing/genetics/software/haplin/or the HAPLIN help pages in ***R***), which automates the analysis of a long sequence of single SNPs, or alternatively a sequence of overlapping sliding-windows with haplotypes. Overlapping sliding-windows will in principle increase the chance of “bracketing=" a causal variant by having a haplotype with SNPs on each side of the variant. However, estimating haplotypes entails a certain loss of power due to the higher number of alleles taken into account and the unknown phase of the haplotypes. It is *a priori* not obvious whether a single-SNP approach or a sliding-window haplotype approach would have the best chance of detecting an association; therefore, we performed both single-marker and haplotype analyses on the current data. We restricted HAPLIN to use up to 4 SNPs in a sliding-window haplotype analysis, which typically ensures bracketing causal variants with two SNPs on each side. Using more than 4 SNPs in a haplotype would most likely entail an unnecessary loss of power. With longer haplotypes, the number of possible haplotypes increases exponentially, and any given haplotype will be found in very few individuals (if any), making effect estimation difficult. After running a sliding-window analysis, the results were summarized in the form of a single, overall p-value associated with each gene. This is done by choosing the smallest p-value from the series of windows. If the tests from each window were independent, this p-value could be adjusted with a standard Bonferroni or Šidák correction. However, when analyzing SNPs in strong LD, and in particular when analyzing overlapping windows of length four haplotypes (which share three SNPs with the neighboring haplotype), there is a strong correlation between results obtained from nearby SNPs or windows, and a Bonferroni correction would be far too strict. The suest function in HAPLIN corrects the minimum p-value for the dependencies in an optimal manner. This is achieved by first saving the individual (family) score contributions from each window estimation. Under the null hypothesis of no disease association with the haplotypes within a window, the score contributions in that window follow an approximate multivariate normal distribution with mean zero and a covariance matrix which can be computed from the estimated individual score contributions. This allows computing the standard score p-value for that window using a chi-squared test statistic [42]. Then, over a series of windows, the combined score contributions from each window follow approximately a multivariate normal distribution with mean zero and an (empirical) covariance matrix computed from the combined score values. This allows computing the theoretical null distribution of the minimum p-value and thus in calibrating (correcting) the observed minimum p-value. This approach is closely related to the principle of “seemingly unrelated estimation=", as implemented in the statistical package Stata [43].

For the second part, QQ plots were used to inspect visually whether our analyses produced more significant results than what would be expected by chance. The rationale behind the procedure is that if no genes have an effect, the computed p-values should derive from a uniform distribution and thus follow the straight diagonal in the QQ plot. If some genes have an effect (with correspondingly lower p-values), their p-values are likely to show up in the QQ plot as a clear deviation from the diagonal, exhibiting a higher significance than expected by chance under the null hypothesis. We generated QQ plots for each cleft type (iCL/P and iCPO) after combining p-values from the Norwegian and Danish HAPLIN analyses using Fisher’s method [44]. The pQQ function in HAPLIN includes confidence bands to assess the size of any deviations from the diagonal. The confidence bands are computed under the null hypothesis of no association between genes and disease. In that case, the order statistic of the sorted p-values will follow a beta distribution with parameters determined by the number of assessed p-values, and the 2.5 and 97.5 percentiles from the corresponding beta distribution provide lower and upper limits, respectively.

#### Software

HAPLIN version 4.1 is implemented in the publicly available ***R*** statistical package [45] and is freely downloadable from our web site at http://www.uib.no/smis/gjessing/genetics/software/haplin. A user-friendly graphical user interface (GUI), which includes some (but not all) of the HAPLIN functionalities, is also freely available at http://haplin.fhi.no.

### Study Approval

Clinicopathological information from all participating families and biologic specimens for DNA extraction were obtained with the written informed consent of the mothers and fathers. The study was approved by the Norwegian Data Inspectorate, the Regional Committee on Research Ethics for Western Norway, and the respective Institutional Review Boards of the US National Institute of Environmental Health Sciences (NIH/NIEHS) and the University of Iowa. For the Danish orofacial clefts study, study approval was obtained from the regional scientific-ethical committee. All aspects of this research are in compliance with the tenets of the *Declaration of Helsinki* for human research (http://www.wma.net).

## Results

**Figures 1** and **2** display the QQ plots for the analysis of 48 SNPs in 18 cleft candidate genes on the X chromosome, without stratification by sex. We first tested a multiplicative model assuming the same relative risk for males and females and no X-inactivation (**Model 2** in **Table 3**). The analyses in **Figure 1** were performed one SNP at a time, whereas **Figure 2** shows the results of the sliding-window haplotype analysis of up to 4 SNPs per window. Overall, the QQ plots show only weak evidence of an association with the oral-facial-digital syndrome 1 gene (*OFD1*, formerly known as *CXORF5*) on chr Xp22 in the Danish iCL/P samples. This association was not replicated in the Norwegian iCL/P samples. For iCPO, there was no evidence of association in either population.

**Figure 3:**
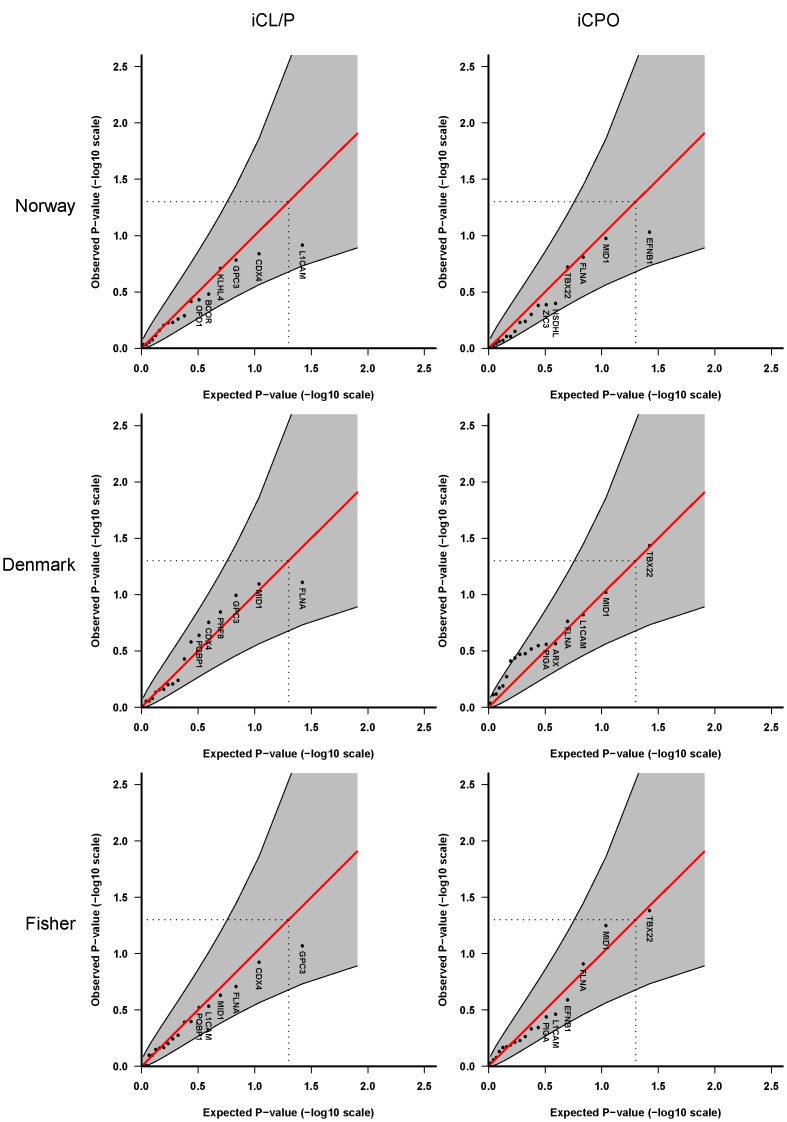
Haplotype analyses of female cases only using up to 4 SNPs per sliding-window. These sex-specific analyses are based on **Model 3** in which we assume different baseline risks for males and females, different relative risks for males and females, and no X-inactivation.

**Figure 4:**
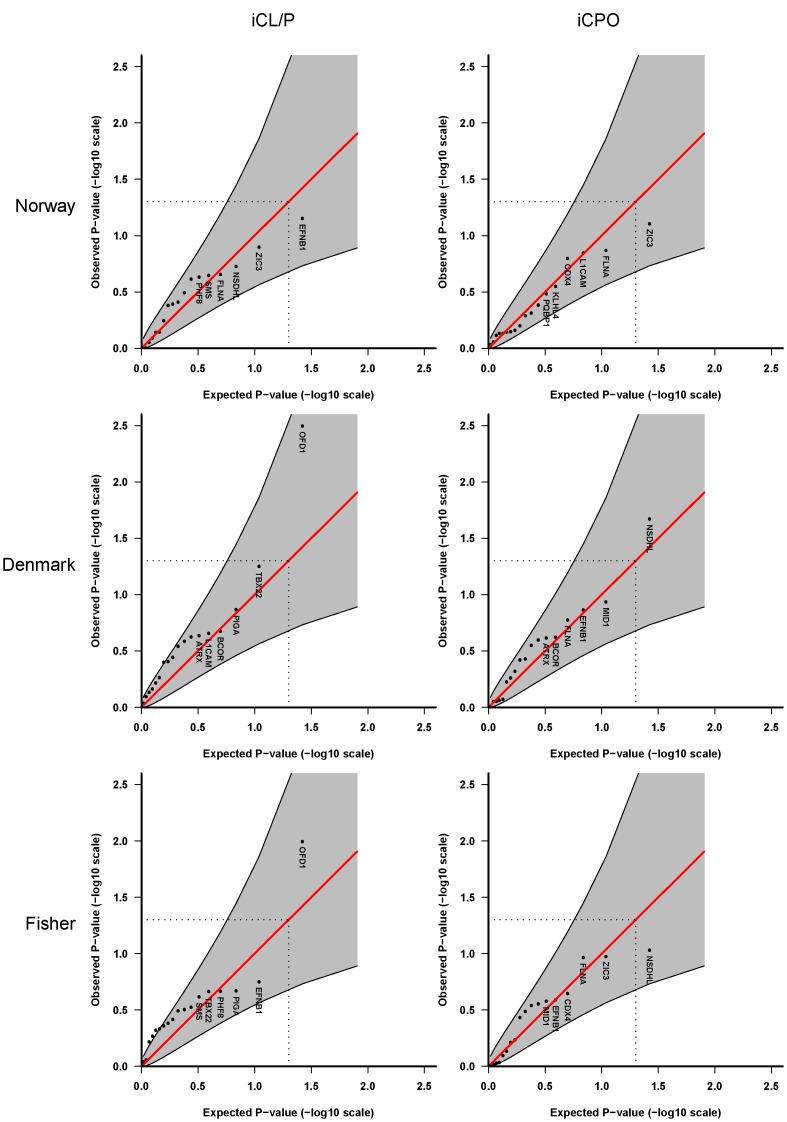
Haplotype analyses of male cases only using up to 4 SNPs per sliding-window, Model 3.

To assess for different gene-effects in males versus females, with effects more evident in one sex stratum than the other, we repeated the analyses for males and females separately. **Figures 3** and **4** show the results of haplotype analysis using a sliding-window of up to 4 SNPs. These analyses correspond to **Model 3** in **Table 3**, in which we assume a multiplicative model with different baseline risks for males and females, different relative risks for males and females, and no X-inactivation. The corresponding single-marker analyses for female and male cases are provided in the online **Figures S1** and **S2**, respectively. The association with *OFD1* is now only observed in the Danish iCL/P males, with a relative risk of 2.2 (95% confidence interval: 1.3–3.7; p-value: 3.6×10^−3^) with one copy of the variant (minor) allele at the *OFD1* SNP rs2285635 when compared with the reference (major) allele.

**Figure 5:**
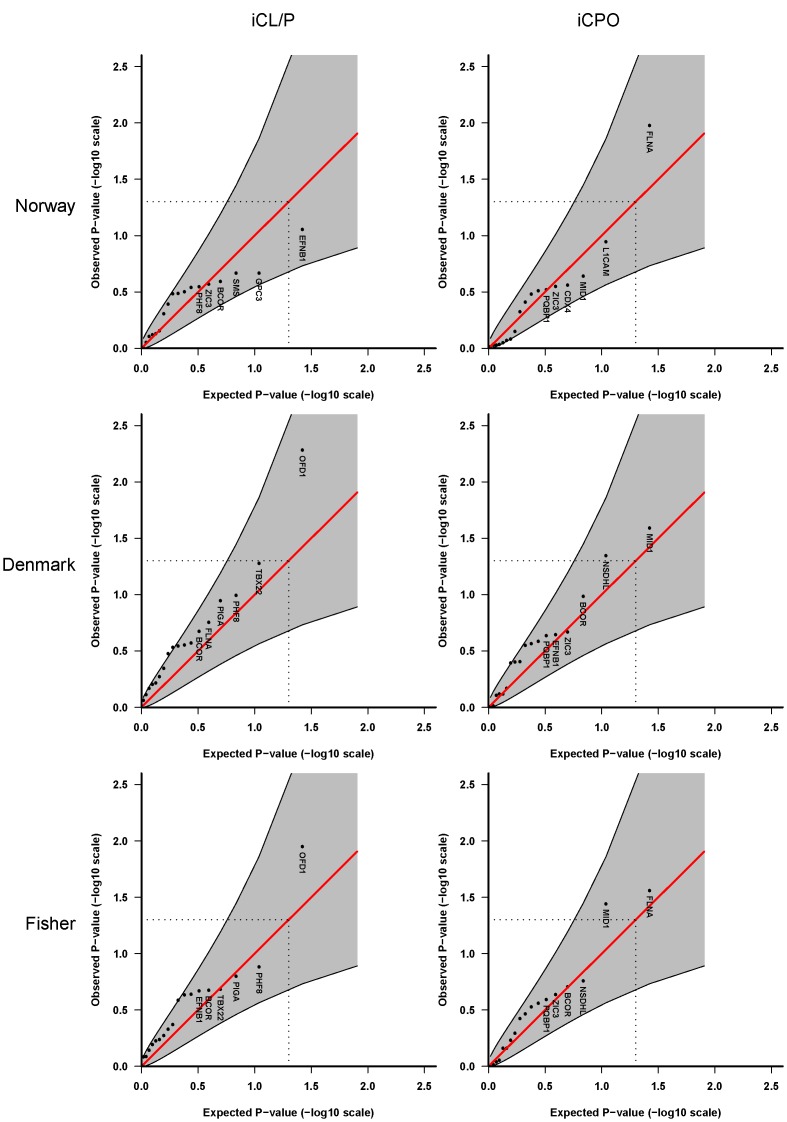
Haplotype analyses using up to 4 SNPs per sliding-window and taking X-inactivation into account. These analyses are based on **Model 4** in which we assume different baseline risks for males and females, a shared relative risk for males and females, and X-inactivation.

Lastly, we tested **Model 4** in which we assume different baseline risks for males and females, a shared relative risk for males and females, and X-inactivation in females. The results of haplotype analysis are depicted in **Figure 5** and the corresponding single-marker analyses are shown in the online **Figure S3**. Again, the only notable association is with *OFD1* in the Danish iCL/P sample only.

## Discussion

Our study was strongly motivated by the unequal sex distribution observed in the two main types of clefts (CL/P and CPO) as well as previous findings of a strong link between X-linked genes and orofacial clefts. X-linked genes have been identified primarily in syndromic forms of clefting and include midline 1 (*MID1*) on chr Xp22, T-box 22 (*TBX22*) on chr Xq21.1, PHD finger protein 8 (*PHF8*) on chr Xp11.22, and RNA binding motif protein 10 (*RBM10*) on chr Xp11.23. Mutations in *MID1* cause the X-linked Opitz GBBB syndrome (OSX, MIM 300000), a congenital midline malformation syndrome characterized by clefting of the lip/palate and a variety of other pathologies [46]. An association between specific haplotypes in *MID1* and isolated CL/P was later reported in an Italian population [19]. Mutations in *TBX22* cause the rare X-linked syndrome ‘cleft palate with ankyloglossia’ (CPX; MIM 303400) [47]. *TBX22* belongs to the T-box family of genes that are evolutionarily highly conserved and recognized for playing key roles in early vertebrate development. Consistent with the CPX phenotype in humans [47]–[49], the expression of *Tbx22* in mice is localized to the developing palatal shelves and the base of the tongue. Further, a genome-wide linkage analysis of families with iCL/P identified a susceptibility locus near *TBX22*, suggesting that the linkage signal may emanate from this gene [50]. Mutations in *TBX22* have also been identified in patients with isolated CPO [51], [52]. As to *PHF8*, mutations in this gene cause the X-linked mental retardation syndrome Siderius that includes cleft palate as a common phenotypic feature [53], [54]. PHF8 is a histone lysine transcription activator expected to have a wide range of functions. Finally, deep sequencing of exons on the X chromosome identified *RBM10* as the gene causing TARP (MIM 311900), a syndromic form of cleft palate [55].

Given these strong links between X-linked genes and syndromic clefts, we examined whether variants in X-linked genes might also be relevant for isolated forms of clefting. To enable X-linked gene analysis, we first developed a method that can i) perform both single-marker and haplotype analyses, ii) generate relevant relative risk estimates with confidence intervals, and iii) assess several etiological models relevant to an X-linked disease locus. The higher prevalence/penetrance for CL/P in males compared with females may be due to hemizygosity for an X-linked disease locus [19]. Therefore, we first analyzed males and females together to account for the possibility that an X-linked disease locus might contribute to clefting risk in both sexes, followed by sex-stratified analyses to investigate whether the X-linked disease locus affects one sex in particular. X-chromosome inactivation in females was also taken into account in the models by treating a heterozygous female (X_1_X_2_) as the average of the two homozygotes (X_1_X_1_ and X_2_X_2_).

Overall we found only weak associations with *OFD1* in the Danish iCL/P sample, with no replication in the Norwegian iCL/P sample. As noted in our previous analyses of fetal gene-effects in the same study samples [32], the genotype call rates for the Norwegian sample (DNA extracted from blood) and Danish sample (DNA extracted from buccal swabs) were 99.6% and 99.1% respectively. Hence, the lack of replication of the *OFD1* association in the Norwegian iCL/P samples cannot be ascribed to differences in DNA source. Moreover, different genotype frequencies do not imply differences in gene effects on the phenotype.

In sex-stratified analyses, the association of *OFD1* in the Danish iCL/P sample was confined to males only, suggesting a possible sex-specific effect as previously reported for several loci when only males were analyzed [56]. Separate analyses for males and females can be potentially more powerful than a pooled analysis if the X-linked disease locus affects only one sex [25]. An alternative explanation for the apparent sex-specific effect in our data is the potentially higher statistical power to detect an effect of *OFD1* in males due to the larger number of male iCL/P cases available for analysis.

Mutations in *OFD1* underlie the X-linked dominant oral-facial-digital syndrome type 1 (OFD1, MIM 311200), which is characterized by malformations of the face, oral cavity and digits, as well as lethality in the vast majority of affected males [57]. Featuring prominently among the orofacial abnormalities are median cleft lip, clefts of alveolar ridge at the area of lateral incisors, and cleft palate [58]. To our knowledge, however, no genetic association with this gene has previously been reported in isolated clefts.

For haplotype analysis of X-chromosome markers, the standard log-linear approach needs some modification. First, many diseases show markedly different birth prevalences in males versus females, as is the case for orofacial clefts, with higher prevalence of CL/P in males and higher prevalence of CPO in females [59]. This difference may be due to causes other than the effect of the particular locus under study, such as loci differentially expressed between males and females. To avoid confounding of the genetic risk estimation by the sex effect, separate baseline risks should be assumed for males and females; i.e. in males the effect of an allele A_2_ should be measured relative to the reference allele A_1_ in males, whereas in females the effects of A_1_A_2_ and A_2_A_2_ should be measured relative to A_1_A_1_ in females (**Table 3**). Second, it is not clear *a priori* whether a single dose of A_2_ in males has an effect comparable to a single dose in females (A_1_A_2_) or to a double dose (A_2_A_2_), or is entirely different from the effect in females. This is aptly illustrated by craniofrontonasal syndrome (CFNS; MIM 304110), an X-linked developmental disorder that paradoxically affects heterozygous females more severely than hemizygous males [60]. In addition, there is the usual question of the relationship between A_1_A_2_ and A_2_A_2_ in females; i.e. whether there is a dose-response relationship, a dominant relationship etc. Third, the basic log-linear model in HAPLIN assumes the same allele frequencies for males and females in the background population. While this is a relatively robust assumption for autosomal markers, it is less obvious for X-linked markers. For populations that are genetically relatively homogeneous (like the Danes and Norwegians), however, this assumption seems to be reasonable.

The most extreme solution to the problems raised above is to run separate analyses on males and females. HAPLIN has a special option for doing this, which allows several different response patterns in females to be explored, whereas males are obviously restricted to single-dose effects. To increase statistical power, HAPLIN allows joint analyses of males and females, which reduce the number of parameters to be estimated. The analyses all assume the same allele/haplotype frequencies but different baseline risks for males and females. In addition, various response patterns can be specified.

Another important consideration is X-inactivation in females which may produce a special relationship between male and female allele effects. In females, one X allele in each cell is inactivated (except for a very few second X chromosomes that escape inactivation). A deleterious X-linked allele would be expected to be more detrimental to males than females because males have no chance of compensation by a corresponding normal allele [61]. Because X-inactivation in women occurs in early embryogenesis, women will tend to have a mixture of cells expressing either their mother’s or father’s X-linked genes (mosaicism). This heterogeneity can have different consequences on a female’s disease response depending on how the two X chromosomes are distributed among tissues [61]. The normal expectation would be an equal distribution of the two cell types [61]. However, there may be “founder=" effects due to the relatively small number of cells in the embryo at the time of X-inactivation, or differential cellular reproduction rates, leading to an imbalance between the two cell types.

If the risk associated with allele A_2_ in females is RR_F_, genotypes A_1_A_1_ and A_2_A_2_ will produce risks B_F_ and B_F_RR_F_, respectively. Assuming a 50∶50 cell type distribution, the risk associated with genotype A_1_A_2_ will be an average of the two homozygotes, i.e. (B_F_+B_F_ RR_F_)/2 = B_F_(1+RR_F_)/2 (**Model 4** in **Table 3**). Technically speaking this is not a log-linear model, so HAPLIN replaces the heterozygous risk with B_F_√RR_F_–the geometric mean of the two homozygous risks. This results in a log-linear model, and as long as RR_F_ is neither very small nor very large, the approximation is reasonable. For males, the single-dose effect is then assumed equal to female homozygotes, i.e. RR_M_ = RR_F_ (denoted simply as RR in **Model 4**). HAPLIN also provides an extension of this model to accommodate an unbalanced cell type distribution.

The basic likelihood models in X-LRT [27] and HAPLIN are similar; for a single SNP, X-LRT uses zero-dose males as reference and estimates relative risks for single-dose males, and zero-, single-, and double-dose females independently. This corresponds to **Model 5** in HAPLIN, and in this special case the results are identical, except that HAPLIN chooses reference levels differently. In addition, HAPLIN provides a number of other modeling options on the X-chromosome, and the software provides a full framework for autosomal and X-linked haplotype association analyses in a candidate-gene or GWAS setting.

To summarize, this is the first candidate-gene based study to investigate the role of X-linked genes in orofacial clefting. Although *OFD1* is a highly plausible gene for clefts, the lack of replication in the Norwegian iCL/P sample highlights the need to confirm these preliminary findings in other datasets. The novel methods presented here address several scenarios relevant to an X-disease locus and can easily be adapted to explore the role of X-linked genes in other complex disorders.

## Supporting Information

Figure S1 **Single-marker analyses of female cases only.** These sex-specific analyses are based on **Model 3** in which we assume different baseline risks for males and females, different relative risks for males and females, and no X-inactivation.
(TIF)

Figure S2 **Single-marker analyses of male cases only, Model 3.**
(TIF)

Figure S3 **Single-marker analyses taking X-inactivation into account.** These analyses are based on **Model 4** in which we assume different baseline risks for males and females, a shared relative risk for males and females, and X-inactivation.
(TIF)

Table S1 **Summary of the 18 X-linked cleft candidate genes and 48 SNPs analyzed in this study.**
(DOC)
