# Supplementary figures and images for: X-Linked Genes and Risk of Orofacial Clefts: Evidence from Two Population-Based Studies in Scandinavia
Source: PLoS One. 2012 Jun 19;7(6):e39240. doi: 10.1371/journal.pone.0039240 (PMC3378529; doi:10.1371/journal.pone.0039240)

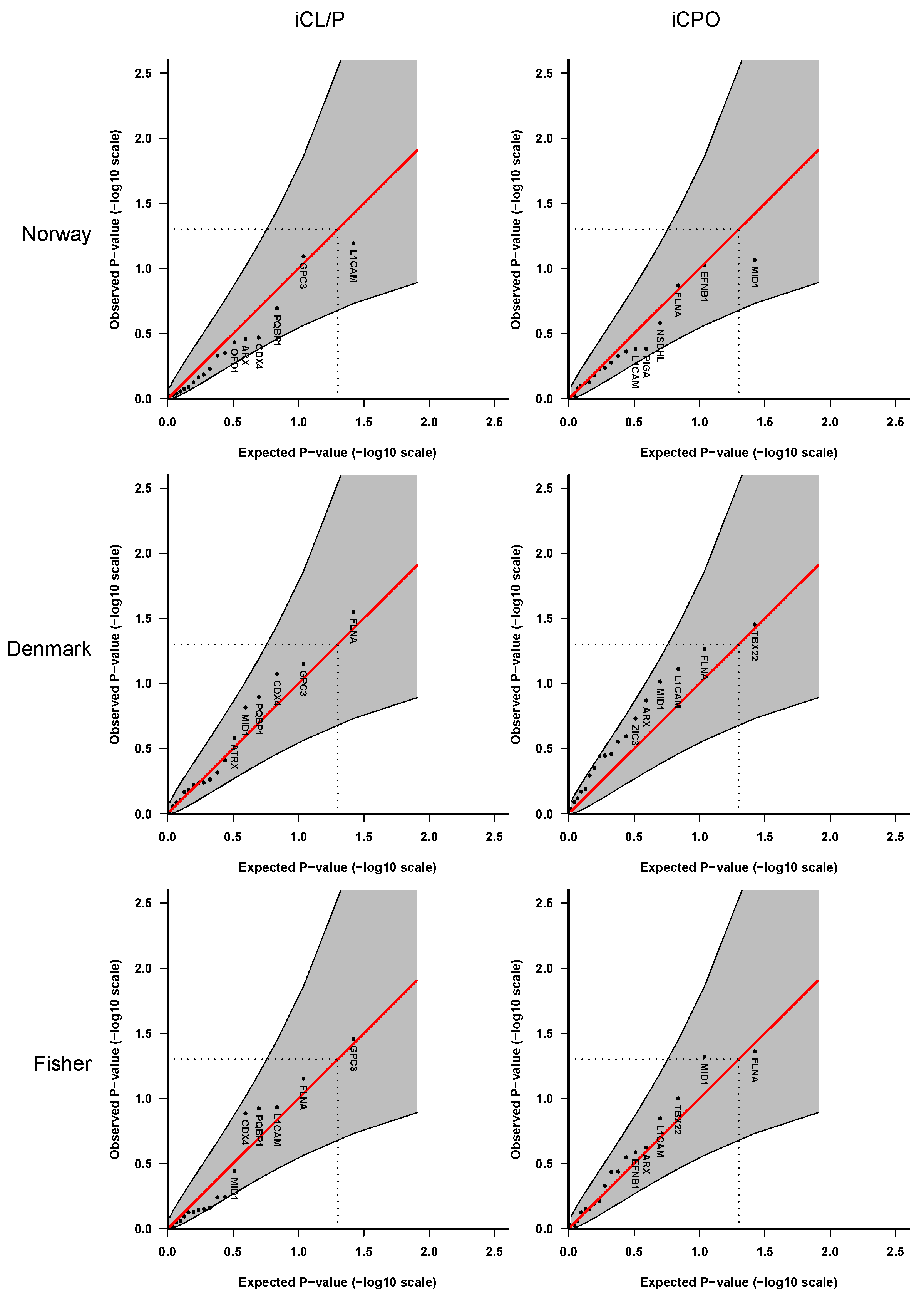

Supplement: Figure S1 — Single-marker analyses of female cases only. These sex-specific analyses are based on Model 3 in which we assume different baseline risks for males and females, different relative risks for males and females, and no X-inactivation. (TIF) [file pone.0039240.s001.tif]

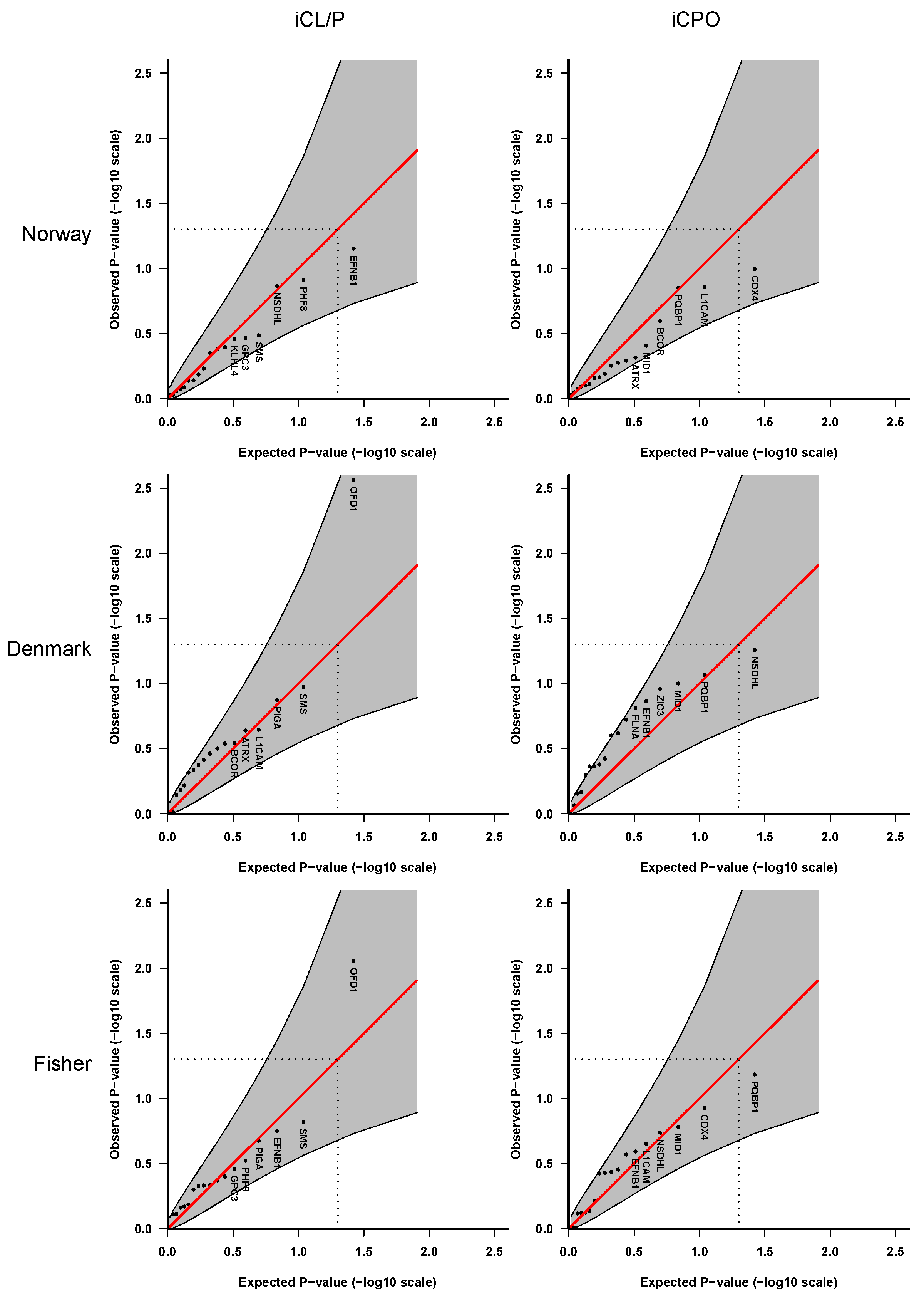

Supplement: Figure S2 — Single-marker analyses of male cases only, Model 3. (TIF) [file pone.0039240.s002.tif]

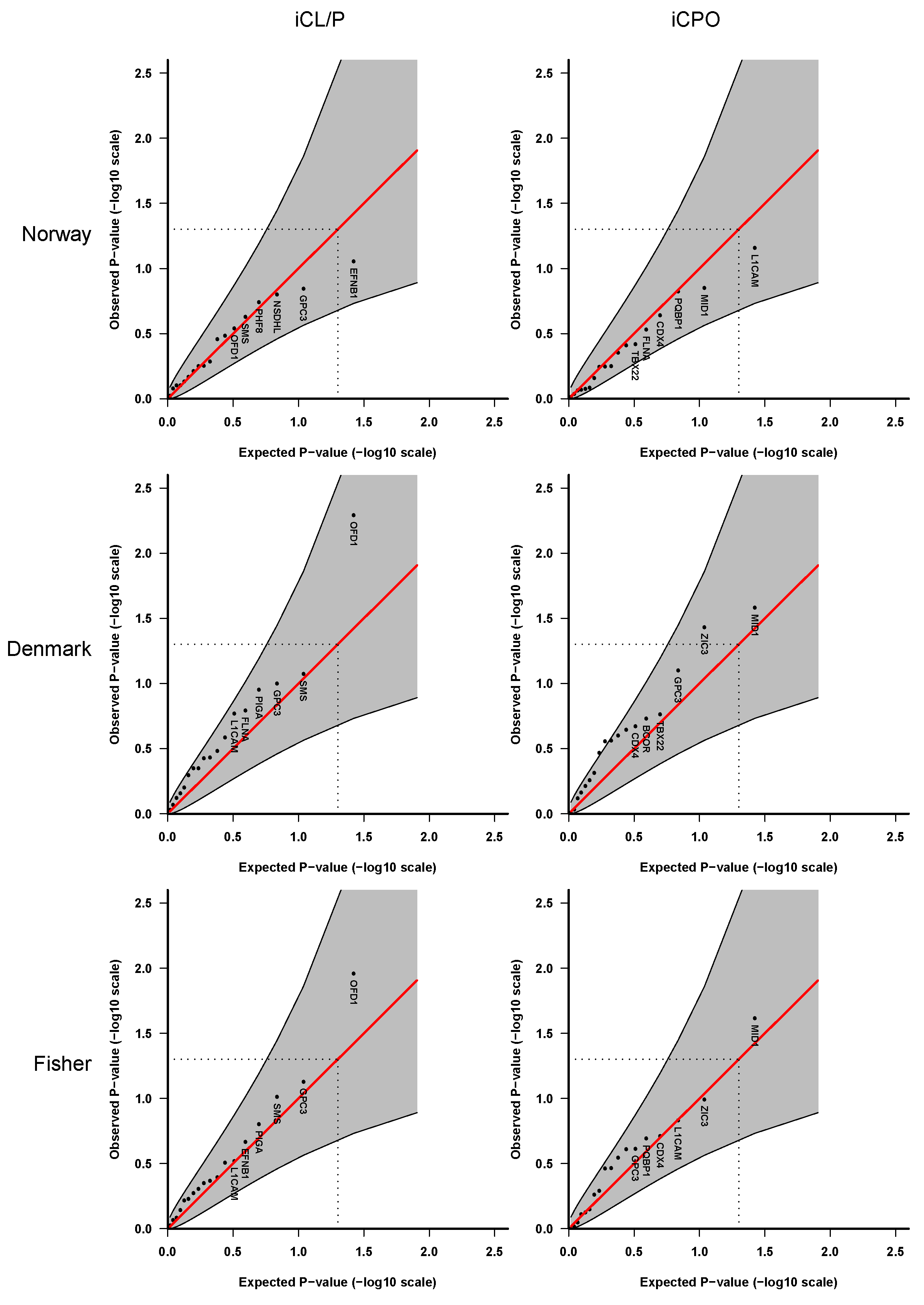

Supplement: Figure S3 — Single-marker analyses taking X-inactivation into account. These analyses are based on Model 4 in which we assume different baseline risks for males and females, a shared relative risk for males and females, and X-inactivation. (TIF) [file pone.0039240.s003.tif]
