# Supplementary material for: X-Linked Genes and Risk of Orofacial Clefts: Evidence from Two Population-Based Studies in Scandinavia
Source: PLoS One. 2012 Jun 19;7(6):e39240. doi: 10.1371/journal.pone.0039240 (PMC3378529; doi:10.1371/journal.pone.0039240)
Supplement: Table S1 — Summary of the 18 X-linked cleft candidate genes and 48 SNPs analyzed in this study. (DOC) [file pone.0039240.s004.doc]

# Tables

**Supplementary Table 1.** Summary of the 18 X-linked cleft candidate genes and 48 SNPs analyzed in this study.

| **Gene ID** | **Gene name ^a^** | **Location** | **Gene Description ^a^** | **SNPs** |
| --- | --- | --- | --- | --- |
| *ARX* | Aristaless related homeobox | Xp21.3 | This is a homeobox-containing gene expressed during development. The expressed protein contains two conserved domains, a C-peptide (or aristaless domain) and the prd-like class homeobox domain. It is a member of the group-II aristaless-related protein family whose members are expressed primarily in the central and/or peripheral nervous system. This gene is thought to be involved in CNS development. Mutations in this gene cause X-linked mental retardation and epilepsy. | rs2285563  rs7063687 |
| *ATRX* | Alpha thalassemia/mental retardation syndrome X-linked | Xq21.1 | The protein encoded by this gene contains an ATPase/helicase domain, and thus it belongs to the SWI/SNF family of chromatin remodeling proteins. The mutations in this gene are associated with an X-linked mental retardation (XLMR) syndrome most often accompanied by alpha-thalassemia (ATRX) syndrome. These mutations have been shown to cause diverse changes in the pattern of DNA methylation, which may provide a link between chromatin remodeling, DNA methylation, and gene expression in developmental processes. This protein is found to undergo cell cycle-dependent phosphorylation, which regulates its nuclear matrix and chromatin association, and suggests its involvement in the gene regulation at interphase and chromosomal segregation in mitosis. Multiple alternatively spliced transcript variants encoding distinct isoforms have been reported. | rs4826225 |
| *BCOR* | BCL6 co-repressor | Xp11.4 | The protein encoded by this gene was identified as an interacting co-repressor of BCL6, a POZ/zinc finger transcription repressor that is required for germinal center formation and may influence apoptosis. This protein selectively interacts with the POZ domain of BCL6, but not with eight other POZ proteins. Specific class I and II histone deacetylases (HDACs) have been shown to interact with this protein, which suggests a possible link between the two classes of HDACs. Several transcript variants encoding different isoforms have been found for this gene. A pseudogene of this gene is found on chromosome Y. | rs6609051  rs12687359 |
| *CDX4* | Caudal type homeobox 4 | Xq13.2 | Not available. | rs1554917  rs2812027 |
| *CXORF5 (a.k.a. OFD1)* | Oral-facial-digital syndrome 1 | Xp22 | This gene encodes a centrosomal protein. A knockout mouse model has been used to study the effect of mutations in this gene. The mouse gene is also located on the X chromosome. However, unlike the human gene, it is not subject to X inactivation. Mutations in this gene are associated with oral-facial-digital syndrome type I and Simpson-Golabi-Behmel syndrome type 2. Many pseudogenes have been identified; a single pseudogene is found on chromosome 5 while as many as fifteen have been found on the Y chromosome. Alternatively spliced transcripts have been described for this gene but the biological validity of these transcripts has not been determined. | rs2285635  rs2283707 |
| *EFNB1* | Ephrin B1 | Xq12 | The protein encoded by this gene is a type I membrane protein and a ligand of Eph-related receptor tyrosine kinases. It may play a role in cell adhesion and function in the development or maintenance of the nervous system. | rs877818 |
| *FLNA* | Filamin A, alpha | Xq28 | The protein encoded by this gene is an actin-binding protein that crosslinks actin filaments and links actin filaments to membrane glycoproteins. The encoded protein is involved in remodeling the cytoskeleton to effect changes in cell shape and migration. This protein interacts with integrins, transmembrane receptor complexes, and second messengers. Defects in this gene are a cause of several syndromes, including periventricular nodular heterotopias (PVNH1, PVNH4), otopalatodigital syndromes (OPD1, OPD2), frontometaphyseal dysplasia (FMD), Melnick-Needles syndrome (MNS), and X-linked congenital idiopathic intestinal pseudoobstruction (CIIPX). Two transcript variants encoding different isoforms have been found for this gene. | rs766419  rs2070822  rs2070816 |
| *GPC3* | Glypican 3 | Xq26.1 | Cell surface heparan sulfate proteoglycans are composed of a membrane-associated protein core substituted with a variable number of heparan sulfate chains. Members of the glypican-related integral membrane proteoglycan family (GRIPS) contain a core protein anchored to the cytoplasmic membrane via a glycosyl phosphatidylinositol linkage. These proteins may play a role in the control of cell division and growth regulation. The protein encoded by this gene can bind to and inhibit the dipeptidyl peptidase activity of CD26, and it can induce apoptosis in certain cell types. Deletion mutations in this gene are associated with Simpson-Golabi-Behmel syndrome, also known as Simpson dysmorphia syndrome. Alternative splicing results in multiple transcript variants. | rs6634941  rs2284125  rs5977928  rs1264380 |
| *KLHL4* | Kelch-like 4 | Xq21.3 | This gene encodes a member of the kelch family of proteins, which are characterized by kelch repeat motifs and a POZ/BTB protein-binding domain. It is thought that kelch repeats are actin binding domains. However, the specific function of this protein has not been determined. Alternative splicing of this gene results in two transcript variants encoding different isoforms. | rs222084  rs222108  rs222110 |
| *L1CAM* | L1 cell adhesion molecule | Xq28 | The protein encoded by this gene is an axonal glycoprotein belonging to the immunoglobulin supergene family. The ectodomain, consisting of several immunoglobulin-like domains and fibronectin-like repeats (type III), is linked via a single transmembrane sequence to a conserved cytoplasmic domain. This cell adhesion molecule plays an important role in nervous system development, including neuronal migration and differentiation. Mutations in the gene cause three X-linked neurological syndromes known by the acronym CRASH (corpus callosum hypoplasia, retardation, aphasia, spastic paraplegia and hydrocephalus). Alternative splicing of a neuron-specific exon is thought to be functionally relevant. | rs5987171  rs4646263  rs3761531  rs5945361 |
| *MID1* | Midline 1 (Opitz/BBB syndrome) | Xp22 | The protein encoded by this gene is a member of the tripartite motif (TRIM) family, also known as the 'RING-B box-coiled coil' (RBCC) subgroup of RING finger proteins. The TRIM motif includes three zinc-binding domains, a RING, a B-box type 1 and a B-box type 2, and a coiled-coil region. This protein forms homodimers which associate with microtubules in the cytoplasm. The protein is likely involved in the formation of multiprotein structures acting as anchor points to microtubules. Mutations in this gene have been associated with the X-linked form of Opitz syndrome, which is characterized by midline abnormalities such as cleft lip, laryngeal cleft, heart defects, hypospadias, and agenesis of the corpus callosum. This gene was also the first example of a gene subject to X inactivation in human while escaping it in mouse. Multiple different transcript variants are generated by alternate splicing; however, the full-length nature of some of the variants has not been determined. | rs741499  rs2525073  rs1965009  rs7886985  rs7053320  rs6530404 |
| *NSDHL* | NAD(P) dependent steroid dehydrogenase-like | Xq28 | The protein encoded by this gene is localized in the endoplasmic reticulum and is involved in cholesterol biosynthesis. Mutations in this gene are associated with CHILD syndrome, which is an X-linked dominant disorder of lipid metabolism with disturbed cholesterol biosynthesis, and typically lethal in males. Alternatively spliced transcript variants with differing 5' UTR have been found for this gene. | rs3788741  rs4828729  rs2071256 |
| *PHF8* | PHD finger protein 8 | Xp11.22 | The protein encoded by this gene is a histone lysine demethylase that preferentially acts on histones in the monomethyl or dimethyl states. The encoded protein requires Fe(2+) ion, 2-oxoglutarate, and oxygen for its catalytic activity. Four transcript variants encoding different isoforms have been found for this gene. An important function of PHF8 is in midline formation and in the development of cognitive abilities, and inactivating mutations result in Siderius type (MRXSSD; OMIM 300263) X linked mental retardation associated with cleft lip/palate. | rs6521788  rs12115965  rs7876951  rs5960612 |
| *PIGA* | Phosphatidylinositol glycan anchor biosynthesis, class A | Xp22.1 | This gene encodes a protein required for synthesis of N-acetylglucosaminyl phosphatidylinositol (GlcNAc-PI), the first intermediate in the biosynthetic pathway of GPI anchor. The GPI anchor is a glycolipid found on many blood cells and which serves to anchor proteins to the cell surface. Paroxysmal nocturnal hemoglobinuria, an acquired hematologic disorder, has been shown to result from mutations in this gene. Alternate splice variants have been characterized. A related pseudogene is located on chromosome 12. | rs5978726 |
| *PQBP1* | Polyglutamine binding protein 1 | Xp11.23 | This gene encodes a nuclear polyglutamine-binding protein that is involved with transcription activation. The encoded protein contains a WW domain. Mutations in this gene have been found in patients with Renpenning syndrome 1 and other syndromes with X-linked mental retardation. Multiple alternatively spliced transcript variants that encode different protein isoforms have been described for this gene. | rs4824733  rs2016813  rs741932 |
| *SMS* | Spermine synthase | Xp22.1 | The protein encoded by this gene belongs to the spermidine/spermine synthases family. This gene encodes a ubiquitous enzyme of polyamine metabolism. | rs2040357  rs5951678 |
| *TBX22* | T-box 22 | Xq21.1 | This gene is a member of a phylogenetically conserved family of genes that share a common DNA-binding domain, the T-box. T-box genes encode transcription factors involved in the regulation of developmental processes. Mutations in this gene have been associated with the inherited X-linked disorder, Cleft palate with ankyloglossia, and it is believed to play a major role in human palatogenesis. Alternatively spliced transcript variants encoding different isoforms have been found for this gene. | rs195295  rs195294  rs195293 |
| *ZIC3* | Zic family member 3 heterotaxy 1 | Xq26.2 | This gene encodes a member of the ZIC family of C2H2-type zinc finger proteins. This nuclear protein probably functions as a transcription factor in early stages of left-right body axis formation. Mutations in this gene cause X-linked visceral heterotaxy, which includes congenital heart disease and left-right axis defects in organs. | rs10856541  rs5931172  rs5931174 |

^a^ Information on gene description was collated from NCBI Entrez Gene (<http://www.ncbi.nlm.nih.gov/gene>).

**Table 1.** Review of family-based methods for association analysis of X-chromosome markers.

| **Reference** | **Method** | **Extended name** | **Attributes** |
| --- | --- | --- | --- |
| Ho and Bailey-Wilson | X-TDT | X-linkage transmission/disequilibrium test (TDT) | This is a TDT for linkage on the X chromosome in the presence of linkage disequilibrium (LD). Under H_o_ of no linkage between disease and marker, the number of transmissions of the variant allele in *n* pairs of heterozygous mothers and their affected children has a binomial distribution with mean *n*/2 and variance *n*/4. The test statistic is a Z-score with a continuity correction, and H_o_ is rejected if Z departs significantly from 0.  As with TDT, X-TDT is readily extended to allow the analysis of phenotypically discordant sib pairs if parental genotypes are unavailable (suitable for late-onset diseases). It can also combine sib-pair and case-parent triad analysis to enhance statistical power. |
| Horvath et al. ; Knapp | XS-TDT; XRC-TDT | X-linked sibling TDT; Reconstruction-combined TDT for X-chromosome markers | As X-TDT above, these are tests for linkage between an X-chromosomal marker and a disease in the presence of LD. XS-TDT uses the genotypes of discordant sibships if genotypes are not available from the parents. It divides the siblings into same-sex groups to account for a possible male/female difference in disease prevalence.  XRC-TDT reconstructs parental genotypes from the genotypes of their offspring and corrects for bias that arise from the reconstruction. Data from families in which parental genotypes are available are combined with families in which genotypes of unaffected sib pairs are available. |
| Ding et al. | XPDT; XMCPDT | X-chromosomal pedigree disequilibrium test; Monte Carlo pedigree disequilibrium test for X-linked markers | XPDT tests for LD in the presence of linkage. It can be applied to any pedigree structure. XMCPDT is an extension of XPDT and infers missing parental genotypes using a Monte Carlo sampling approach.  XPDT is limited to same-sex discordant sib pairs when parental data are missing, resulting in lower statistical power. XMCPDT on the other hand requires allele frequency estimates to compensate for missing parental genotypes. XMCPDT appears to have superior power than XSTDT, XRCTDT or XPDT when there are missing data, but Type 1 errors can be inflated when a large proportion of parental genotypes are missing. |
| Chung et al. | X-APL | A modification of the “association in the presence of linkage test (APL)” that accommodates X-chromosome markers | Like XPDT, X-APL can use singleton or multiplex families. The APL statistic is based on difference between the observed versus the expected number of a specific allele in affected siblings conditional on the parents’ genotypes. X-APL infers missing parental genotypes in linkage regions by using identity-by-descent (IBD) parameters for affected siblings.  X-APL can test individual markers or haplotypes. For haplotype tests, X-APL assumes no recombination between the markers within the families in the sample, and the EM algorithm is used for haplotype phase estimation.  X-APL can also perform sex-stratified analyses to account for different penetrance of disease in males versus females. |
| Zhang et al. | X-LRT | A likelihood ratio test of association for X-linked markers. | X-LRT is a likelihood-based method and enables estimation of genetic risks. The method is designed for singleton families but can also allow additional siblings. Missing parental genotypes can be accounted for using the EM algorithm, and even more efficiently using sibling genotype information when available.  For haplotype relative risk estimation, X-LRT assumes no recombination between markers, parental mating to be random, and haplotype penetrance to be multiplicative for females.  For sex-specific analysis, separate risk parameters are introduced for males and females in single-marker analyses, but in haplotype analyses the data are divided into two sets, one containing only male cases and the other only female cases. |
| This paper | HAPLIN | A full likelihood model for haplotype associations at autosomal and X-linked markers. | HAPLIN is a likelihood-based method and enables estimation of genetic risk associated with marker haplotypes both for autosomal and X-linked markers. It applies to case-parent triad data, possibly combined with independent controls and/or complete control-parent triads. Missing data are imputed using the EM algorithm.  On the X chromosome, HAPLIN provides a range of model options depending on haplotype effects in females versus males. A complete sex stratification implies different haplotype frequencies, different baseline risks and different relative risks between males and females. Alternatively, haplotype frequencies can be assumed equal, as can haplotype relative risks. The risk response pattern may depend on the number of risk haplotypes, and X-inactivation in females can be incorporated. |

1. Ho GY, Bailey-Wilson JE (2000) The transmission/disequilibrium test for linkage on the X chromosome. Am J Hum Genet 66: 1158-1160.

2. Horvath S, Laird NM, Knapp M (2000) The transmission/disequilibrium test and parental-genotype reconstruction for X-chromosomal markers. Am J Hum Genet 66: 1161-1167.

3. Knapp M (2001) Reconstructing parental genotypes when testing for linkage in the presence of association. Theoretical population biology 60: 141-148.

4. Ding J, Lin S, Liu Y (2006) Monte Carlo pedigree disequilibrium test for markers on the X chromosome. Am J Hum Genet 79: 567-573.

5. Chung RH, Morris RW, Zhang L, Li YJ, Martin ER (2007) X-APL: an improved family-based test of association in the presence of linkage for the X chromosome. Am J Hum Genet 80: 59-68.

6. Zhang L, Martin ER, Chung RH, Li YJ, Morris RW (2008) X-LRT: a likelihood approach to estimate genetic risks and test association with X-linked markers using a case-parents design. Genet Epidemiol 32: 370-380.

**Table 2.** Assorted parameterization models for analysis of X-linked gene variants using the HAPLIN software.

| Model | Male case | |  | Female case | | |
| --- | --- | --- | --- | --- | --- | --- |
|  | X_1_ | X_2_ |  | X_1_X_1_ | X_1_X_2_ | X_2_X_2_ |
| Model 1 | B | B*RR |  | B | B*RR | B*RR^2^ |
| Model 2 | B_M_ | B_M_*RR |  | B_F_ | B_F_*RR | B_F_*RR^2^ |
| Model 3 | B_M_ | B_M_*RR_M_ |  | B_F_ | B_F_*RR_F_ | B_F_*RR_F_^2^ |
| Model 4 | B_M_ | B_M_*RR |  | B_F_ | 1/2*B_F_*(1 + RR) | B_F_*RR |
| Model 5 | B_M_ | B_M_*RR_M_ |  | B_F_ | B_F_*RR_F1_ | B_F_*RR_F2_ |

X_1_ denotes the common allele and X_2_ the variant or target allele for a given SNP; ‘*’ denotes the product term; B represents the shared baseline risk for males and females; B_M_ is the baseline risk for males only; B_F_ is the baseline risk for females only; RR is the shared relative risk for males and females; RR_M_ is the relative risk for males only; and RR_F_ is the relative risk for females only. In Model 4, the risk for an X_1_X_2_ female is the average of the two homozygotes; i.e. (B_F_ + B_F_*RR)/2 = B_F_(1 + RR)/2. As this is not a log-linear model, HAPLIN replaces the heterozygous risk with B_F_√RR, i.e. the geometric mean of the two homozygous risks. Models 3 and 5 can be estimated assuming equal or unequal haplotype frequencies between males and females.
